# DNA methyltransferases and their roles in tumorigenesis

**DOI:** 10.1186/s40364-017-0081-z

**Published:** 2017-01-20

**Authors:** Wu Zhang, Jie Xu

**Affiliations:** State Key Laboratory for Medical Genomics, Shanghai Institute of Hematology, Rui-Jin Hospital affiliated to Shanghai Jiao-Tong University School of Medicine, 197 Rui Jin Er Road, 200025 Shanghai, China

**Keywords:** DNA methyltransferases, Tumorigenesis, DNA methylation

## Abstract

DNA methylation plays an important role in gene expression, chromatin stability, and genetic imprinting. In mammals, DNA methylation patterns are written and regulated by DNA methyltransferases (DNMTs), including DNMT1, DNMT3A and DNMT3B. Recent emerging evidence shows that defects in DNMTs are involved in tumor transformation and progression, thus indicating that epigenetic disruptions caused by DNMT abnormalities are associated with tumorigenesis. Herein, we review the latest findings related to DNMT alterations in cancer cells and discuss the contributions of these effects to oncogenic phenotypes.

## Background

DNA methylation is one of the most important epigenetic modifications [1], playing key roles in the regulation of gene expression, genomic imprinting, X chromosome inactivation, and tumorigenesis [2, 3]. In mammals, DNMT1, DNMT3A and DNMT3B, the generally recognized three types of DNA methyltransferases (DNMTs), execute the genomic methylation process [4]. These proteins are highly conserved and have similar amino acid sequences. The N-terminus contains a regulatory domain, which allows DNMTs to anchor in the nucleus and recognize nucleic acids or nucleoproteins, and the C-terminus possesses a catalytic domain, which is responsible for the enzymatic activity [5]. DNMT1, DNMT3A and DNMT3B have different functions in the methylation process. DNMT1 is required for the maintenance of all methylation in the genome. During replication, DNMT1 restores the specific methylation pattern on the daughter strand in accordance with that of the parental DNA. DNMT3A and DNMT3B are referred to as *de novo* methyltransferases, which are responsible for establishing DNA methylation patterns during embryogenesis and setting up genomic imprints during germ cell development [6]. Although they are highly expressed in early mammalian embryos, DNMT3A and DNMT3B decrease in expression over the course of cell differentiation. These two proteins have distinct functions throughout embryonic development, showing both spatial and temporal differences. DNMT3A primarily methylates a set of genes and sequences at the late stage of embryonic development and especially after birth, whereas DNMT3B modifies a broader region of genomic sequences in early embryos [2, 6]. Very recently, one study identified a new *de novo* DNA methyltransferase DNMT3C in murine germ cells. DNMT3C exhibits high identity with DNMT3B, and is specialized at methylating the young retrotransposons [7]. Beside the above-mentioned enzymes, which are essential for the methylation of mammalian DNA, the DNMT family also includes two additional members, DNMT2 and DNMT3L. Although DNMT2 is not currently considered to be a DNA methylase, this enzyme methylates small transfer RNAs (tRNAs) [8]. DNMT3L, an important regulator without catalytic activity, operates in the form of DNMT3L-DNMT3A heterotetramers and facilitates the methylation of cytosine residues [2, 5, 6]. In animal models, Dnmt3a knockout mice have been found to exhibit postnatal growth retardation and dysplasia and to die by 4 weeks of age [9]. Mice deficient in either Dnmt1 or Dnmt3b exhibit embryonic lethality [9, 10]. Male mice without Dnmt3c are sterile [7]. Thus, these phenotypes demonstrate that the establishment and maintenance of global genomic methylation processes is the basis for cell proliferation and differentiation.

In recent years, interest in the relationship between DNA methylation and human diseases has increased. Alterations in DNA methylation patterns have been implicated in tumorigenesis in several studies [11–13]. Owing to the revolutionary progress of next-generation sequencing technology, a variety of genomic landscapes of human tumor tissues have been described, and a number of defective genes associated with illnesses have been discovered [4, 13]. Sequencing studies on hematologic disorders achieve big success in identifying previously unrecognized mutated genes [14]. Among these mutated genes, many, such as *DNMT3A*, *TET2*, and *IDH1*, are involved in epigenetic processes [15–18] and are directly or indirectly related to DNA methylation. These discoveries bring new prospects for cancer diagnosis and treatment, enabling researchers to fully realize the enormous potential of genomic methylation abnormalities in tumorigenesis. The following content will describe the relationship between defective DNMTs and tumorigenesis, and finally will focus on the DNMT3A alteration that has been especially well studied.

## Emerging evidence of DNMTs in malignant transformation

Tumor cells typically exhibit aberrant DNA methylation patterns during malignant transformation [3, 19]. Although this phenomenon is generally attributed to different mechanisms, alteration in the DNMT family of genes and the resulting dysregulation of genomic methylation is a primary causative factor [20, 21]. Numerous samples with lesions in the DNMT genes have been studied to identify methylation changes and to evaluate cancer development. These lesions can be classified into three categories: overexpression, mutation and deletion (Table 1).

**Table 1:** Emerging evidence of DNMTs in malignant transformation

Tumor type	DNMT subtype	Model studied	Alteration	Reference
AML	DNMT3A DNMT3A DNMT3A DNMT3B DNMTs	Patients Mouse tumor model Mouse tumor model Mouse tumor model Patients	Mutation Mutation Deletion Deletion Overexpression	[15, 16, 34] [72] [39–43] [46] [74]
MDS	DNMT3A	Patients	Mutation	[35]
CMML	DNMT3A	Mouse model	Mutation	[37]
CML	DNMTs	Patients	Overexpression	[74]
ALL	DNMT3A	Patients	Mutation	[36]
Lymphoma	DNMT1 DNMT3A DNMT3B	Mouse tumor model Mouse model Mouse tumor model	Deletion Deletion Deletion	[47] [44] [75]
Breast	DNMT1 DNMT1 DNMT3B	Mouse tumor model Patients Patients	Deletion Overexpression Overexpression	[76] [77] [26]
Lung	DNMT3A	Mouse tumor model	Deletion	[45]
Colon	DNMT1 DNMT3B DNMT3B	Patients Patients Mouse tumor model	Mutation Overexpression Overexpression	[33] [29, 30] [27, 28]
Liver	DNMT1 DNMT3A	Patients Patients	Overexpression Overexpression	[24] [25]
Melanoma	DNMT3A	Mouse tumor model	Overexpression	[78]
Pancreas	DNMT1	Patients	Overexpression	[23]
Prostate	DNMT3B	Patients	Overexpression	[31]
Esophagus	DNMT1	Patients	Overexpression	[22]

### Overexpression

Overexpression of DNMTs (DNMT1, DNMT3A, and DNMT3B) in a variety of tumors results in hypermethylation and oncogenic activation [11]. DNMT1 overexpression correlates well with aberrant DNA methylation in solid tumors, thus resulting in lymph node metastasis and poor prognosis in patients [22–24]. Similarly, highly expressed DNMT3A or DNMT3B has been found in a large number of patient specimens, and increased DNMT3A expression is involved in hepatocellular carcinogenesis [25]. Moreover, high expression levels of DNMT3B and CTCF are critical in the epigenetic inactivation of *BRCA1* in sporadic breast tumors [26]. Additional studies have suggested that DNMT3B is required for the outgrowth of colonic micro-adenomas [27, 28]. Several studies have provided explanations for the relationship between overexpressed DNMTs and tumorigenesis. Zhao et al. have shown that DNMT1 knockdown has an inhibitory effect on the cell cycle in esophageal squamous cell carcinoma, indicating that increased methylation levels promote cell mitosis [22]. Two groups have demonstrated that DNMT3B overexpression is closely related to CIMP-high in colon cancers [29, 30]. Additional studies performed on cultured primary prostate cells have shown that the overexpression of DNMT3B1 and DNMT3B2, the two subtypes of DNMT3B, leads to an increase in methylation [31].

### Mutation

Somatic mutations in DNMTs are the prominent features of many tumors and substantially contribute to malignant transformation [32]. As shown in Table 1, DNMT1 mutations in colon tumors and DNMT3A mutations in hematological malignancies have been observed in the cancer genome. Kanai et al. have shown that DNMT1 inactivation due to mutational changes in colon cancers results in genome-wide alterations of the DNA methylation status [33]. Critical findings on DNMT3A variation have suggested that DNMT3A is frequently mutated in acute myeloid leukemia (AML), myelodysplastic syndrome (MDS) and adult early T-cell precursor acute lymphoblastic leukemia (ETP-ALL) and is associated with disease aggressiveness and treatment resistance [15, 16, 34–36]. Mice expressing the Dnmt3a Arg882 mutant protein developed chronic myelomonocytic leukemia with thrombocytosis [37]. Moreover, DNMT3A mutations, particularly those in the catalytic domain, substantially decrease enzymatic activity [16, 34]. In DNMT3A-mutated AML samples and relevant mouse models, such loss of function results in the hypomethylation of *HOX* family genes [16, 37]. Together, these studies suggest that mutated DNMTs disrupt genomic methylation and play significant roles in tumor formation.

### Deletion

An in vivo mouse model with embryonically inactive DNMT3A and DNMT3B has shown that the deletion of *de novo* methyltransferases leads to lethal phenotypes [9]. Recently, the effects of *de novo* methyltransferase on hematopoiesis have been evaluated through conditional knockout technology. The deletion of Dnmt3a in adult mice induces the proliferation of hematopoietic progenitors [38]. On the basis of this abnormality, researchers then demonstrated that mutated NRAS- or FLT3-ITD-driven malignancy is accelerated by a lack of Dnmt3a [39–42]. Furthermore, the ectopic introduction of c-Kit variants into a Dnmt3a-deficient background produces acute leukemia [43]. Moreover, DNMT3A inactivation leads to the progression of peripheral T cell lymphoma (PTCL) and lung tumors, thus indicating that *DNMT3A* may act as a tumor-suppressor gene [44, 45]. Studies have also shown that DNMT3B acts as a tumor suppressor in Myc-induced lymphomas and MLL-AF9-driven AML [46]. A lack of maintenance methyltransferase activity is also related to carcinogenesis. Studies have shown that DNMT1 deletion leads to DNA demethylation and that DNMT1 is critical for T-cell lymphoma prevention and maintenance, contributing to aberrant methylation by *de novo* and maintenance methylation [47]. Therefore, deletion of genes encoding DNMTs also participates in tumor development.

## Epigenetic disruptions involving DNMTs in tumorigenesis

**Fig. 1:**
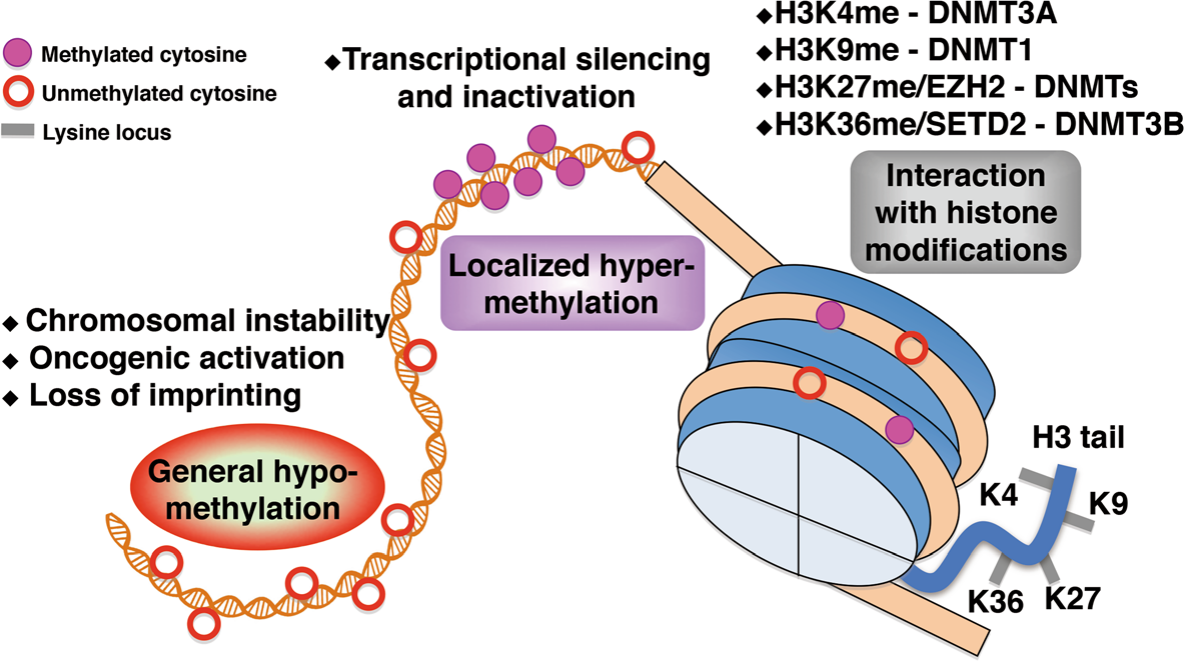
Epigenetic alterations involving DNMTs in tumorigenesis. Numerous clinical and experimental data suggest that tumor cells generally exhibit genome-wide hypomethylation and localized hypermethylation, in contrast with normal cells. Interactions between DNMTs and histone methyltransferases, such as EZH2 and SETD2, play critical roles in epigenetic disruption during malignancy. Thus, the identification of epigenetic alterations involving DNMTs in tumorigenesis may contribute to improved cancer diagnosis and effective treatments

Epigenetic disorders, which are commonly found in cancer, are attributed in part to DNMT dysfunction [3, 4]. Because of its catalytic role and inhibition of target gene transcription, DNMTs play a significant role in the maintenance of chromosomal homeostasis [6]. Defective DNMTs induce imbalances in DNA and/or histone modification, thus resulting in chromatin remodeling, genomic instability and gene inactivation. Unlike the genomes in normal tissue, the genomes of tumor cells generally display global hypomethylation throughout, with localized hypermethylation in particular regions [20]. Moreover, crosstalk between DNMTs and other chromatin regulators, such as histone methyltransferases and transcriptional co-suppressors, is highly important in epigenetic disruption [48–50]. These characteristics may contribute to diagnosis and targeted therapy in clinical applications (Fig. 1).

### Global hypomethylation

DNA hypomethylation of tumor cells is the first process characterized as an epigenetic abnormality [19]. The genome-wide hypomethylation of tumor cells results in a reduction of 5-mC, mainly in gene-coding regions and satellite repeats (Fig. 1). These changes cause mitotic recombination, copy number deletion and chromosomal rearrangement, and even genomic imprinting annihilation. Gaudet et al. have demonstrated that deletion or reduction of DNMT1 leads to substantial genome-wide hypomethylation and chromosomal instability [51]. Through methylated DNA immunoprecipitation (MeDIP)-chip analysis, hypomethylated CpG islands (CGIs) of the *HOXB* cluster have been found in AML samples with *DNMT3A* mutations [16]. Although the underlying mechanism governing the effects of genome-wide hypomethylation on the process of tumorigenesis is not fully understood, these limited data have provided alternative insight into a relationship between aberrant DNMTs and global hypomethylation, along with subsequent tumor occurrence [1].

### Localized hypermethylation

In normal somatic cells, DNA methylation occurs primarily in dinucleotides containing less CpG, whereas the CpG-enriched region is unmethylated [6, 50]. Throughout malignant transformation, the global methylation level of DNA changes, thus leading to non-CpG island hypomethylation and CGI hypermethylation. As a result, the number of genes that are hypermethylated on their promoters increases. In particular, hypermethylation induces the silencing of several key tumor-suppressor genes (TSGs), which play important roles in tumor progression (Fig. 1). Generally, abnormal CGI hypermethylation is an epigenetic characteristic of tumors, of which hypermethylated TSGs are the most common feature [1, 13].

A great deal of research has been performed to explore the mechanism of aberrant TSG methylation in tumor tissue. As expected, DNMTs have been included in the aforementioned studies. In leukemia, deletions or mutations in DNMTs often disrupt the distribution of 5-mC in the genome [52]. Butcher et al. have shown that in some sporadic breast tumors, hypermethylation of the *BRCA1* promoter is partially due to DNMT3B overexpression [26]. Using a conditional Dnmt3a knockout mouse model, researchers have observed a general decrease in hypomethylation in the transcription factor-binding sites of cross-regions (Canyons) [53]. Additionally, canyon-associated genes, including *HOX* genes, are markedly enriched in DNMT3A mutant AMLs [53].

Tumor-associated DNA methylation generally occurs in the promoter regions of TSGs [13]. However, owing to rapid advancements in methylation sequencing, data increasingly indicate that a large number of non-TSGs are methylated at the early stage of tumor initiation, and methylation changes within the gene body have a substantial effect on the process of transcription. The TCGA network has reported the integrated methylation profiles of AML samples with mutations in *DNMT3A*, as determined with Human Methylation 450 Bead Chip arrays [54]. This complete epigenomic landscape reveals a large amount of hypermethylated cytosine bases in the gene body and intergenic regions. Similarly, in a Dnmt3a mutant-transduced mouse model, hypermethylation is greater in the intergenic regions, and a cluster of suppressed genes related to lymphocyte development, such as *Notch1* and *Gata3*, are hypermethylated in the gene body regions [37]. Furthermore, Yang et al. have suggested that DNMT3B-dependent gene body methylation enhances transcription and may be a potential therapeutic target in cancer [55].

### Interaction with histone modifications

The entire epigenetic profile of the genome shows that active chromatin regions are generally characterized by acetylated histones and unmethylated DNA, whereas methylated histones associated with repressed chromatin and methylated DNA are enriched in suppressed regions [50]. Thus, the two chromatin markers interact in a highly orchestrated manner and are closely linked: DNA methylation helps guide histone modification, and histone modification directs DNA methylation (Fig. 1). For example, DNMT1 is required for the maintenance of H3K9 methylation in human cancer cells [56], and DNMT3A PWWP interacts with H3K36me3 and consequently enhances DNMT3A activity [57]. These effects can be regarded as the outcome of cooperation between histone methyltransferase (HMT) and DNMTs. Indeed, DNMTs form complexes with HMTs and consequently regulate transcription. Both the H3K36 methyltransferase SETD2 and the PWWP domain of DNMT3B are required for the *de novo* methylation of transcribed genes [58]. Likewise, the ADD domain of DNMT3A recognizes unmodified H3, which is repressed by H3K4 methylation [59]. In undifferentiated human embryonic carcinoma cells, promoter-related DNMTs overlap with different histone modifications [60]. Two groups have demonstrated that DNMT1 improves genomic methylation through enhanced histone modification by EZH2. EZH2 polycomb group protein mediates H3K27 methylation and recruits and directly controls DNA methylation [61, 62]. Thus, the above-mentioned investigations confirm that abnormal DNA methylation in tumor cells is closely related to histone modification. The relationship between DNA methylation and histone modification should provide more comprehensive insights into epigenetic regulation in tumorigenesis.

## DNMT3A alterations lead to epigenetic reprogramming in leukemia

In recent years, mutated genes encoding a group of epigenetic modification regulators have attracted attention because of their high frequency of variation in hematological diseases [63]. Epigenetic disruption due to genetic alterations is the root cause of malignant transformation, particularly in hematologic malignancies [64]. Most notably, through a variety of high-throughput techniques, somatic mutations involving the *DNMT3A* gene have been identified in AML at a mutation rate of ~20%, and the prognosis for mutant patients is relatively poor [65]. Currently, DNMT3A abnormalities are the most common subject in the field of epigenetic medical research, because of its significance in tumor pathogenesis and the potential for target medication. Herein, the organization characteristic of DNMT3A and critical implications of DNMT3A alterations in hematological cancers are highlighted.

### DNMT3A structure and function

As a member of the DNMT family, DNMT3A possesses the characteristic peptide structure: its catalytic domain directly binds to S-adenosyl-L-methionine (SAM) and DNA strands, and the N-terminus regulation domain is primarily involved in nuclear localization and protein interactions, in which the PWWP domain interacts with methyl lysine histones, and the PHD domain recognizes unmethylated histones. These functions serve as a signal of the histone transfer effect, thus ensuring diverse epigenetic modification [5]. Specifically, DNMT3A forms a butterfly-shaped tetramer (DNMT3L-DNMT3A-DNMT3A-DNMT3L) in the C-terminus with DNMT3L, thus changing the conformation of DNMT3A and facilitating its catalytic activity. The N-terminus of DNMT3A also operates as a transcriptional repressor. The regulatory domain recruits nucleoproteins into the complex and performs histone modifications, chromatin remodeling and gene transcription. A range of partners is known to interact with DNMT3A, including histone methyltransferases, histone deacetylases, and various transcription factors, even enzymes in the DNMT family [50]. DNMTs are bound to each other, and *de novo* methyltransferase said in coordination during methylation maintenance [66]. Studies have shown that H3K9 methyltransferases, such as SUV39H1 and SETDB1, can directly bind to the PHD domain of DNMT3A and improve each other’s catalytic activity, thus indicating that different epigenetic modifications can enhance chromatin inhibition by cooperating together [67].

### DNMT3A mutation in leukemia

**Fig. 2:**
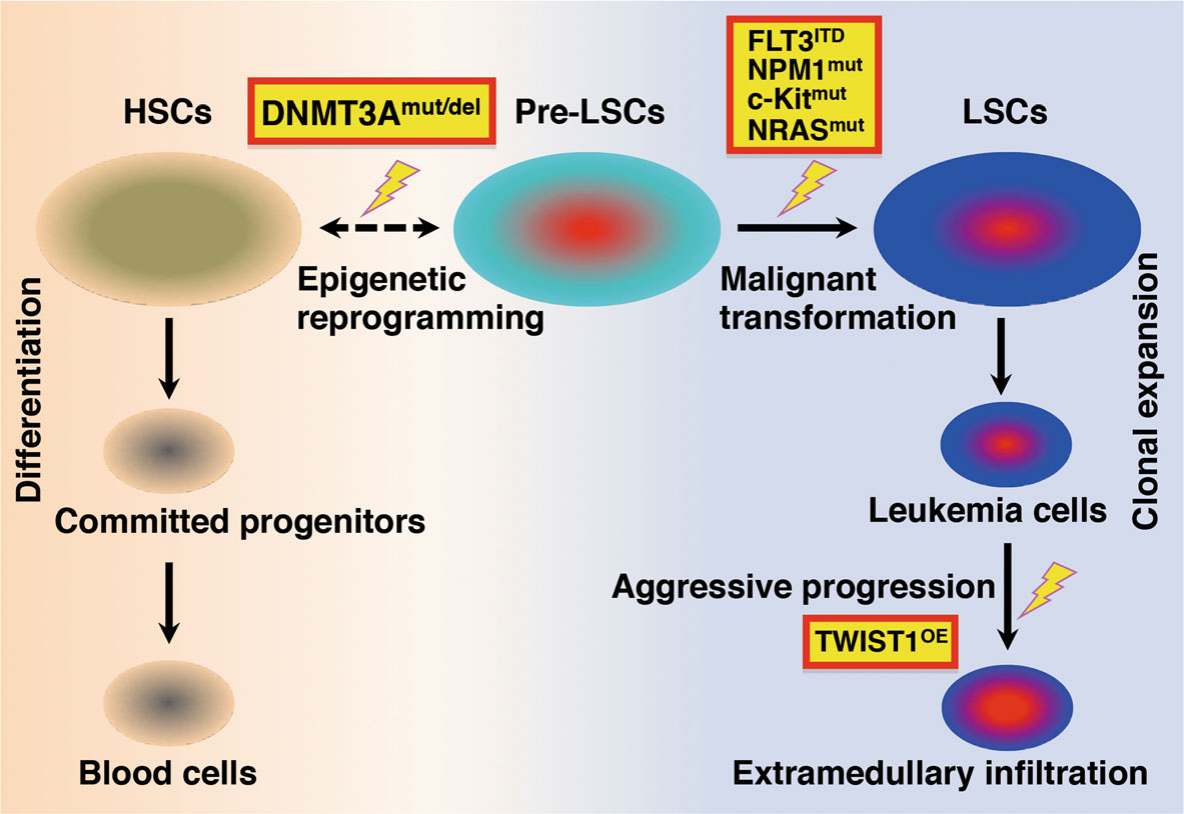
DNMT3A alterations lead to epigenetic reprogramming in leukemia. Leukemia is a heterogeneous disease caused by cumulative multi-step disruption. In the initial stage of leukemogenesis, accumulated DNA lesions, emergent stimuli, and metabolic stress are observed in hematopoietic stem cells (HSCs). These conditions lead to gene alterations and link epigenetic reprogramming to leukemia development. Currently, DNMT3A gene lesions are considered to be critical epigenetic alterations in the occurrence of leukemia. In patient specimens and mouse models, the mutation or deletion of *DNMT3A* causes the apparent reversal of normal HSCs into pre-leukemia stem cells (Pre-LSCs). Frequently, Pre-LSCs are quiescent and stable in the early phases of leukemia. The accumulation of other transformative changes, such as a series of mutations (RAS^mut^, NPM1^mut^, c-Kit^mut^) or oncogenic alterations (FLT3^ITD^) causes Pre-LSCs to undergo malignant transformation into leukemia stem cells (LSCs), which finally enter the clonal expansion stage. Furthermore, during the aggressive progression of leukemia in a xenograft mouse model of OCI-AML3 with mutated DNMT3A, DNMT3A mutation promotes leukemic extramedullary infiltration by up-regulating the expression of the EMT inducer TWIST1. HSCs: hematopoietic stem cells; Pre-LSCs: pre-leukemia stem cells; LSCs: leukemia stem cells; mut: mutation; del: deletion; ITD: internal tandem duplication; OE: overexpression; EMT: epithelial-mesenchymal transition

Clinically, in patients with *DNMT3A* mutations, the number of leukocytes present at diagnosis is relatively higher, and survival is comparatively shorter [15, 16]. To date, numerous functional experiments have provided a better understanding of the effects of *DNMT3A* mutation on leukemia pathogenesis (Fig. 2). For instance, *DNMT3A* mutation is an early event in the initiation of hematopoietic disorders and is one of several causative factors for the establishment of founder clones and the transformation of hematopoietic stem cells (HSCs) to pre-leukemic stem cells (Pre-LSCs) [68]. In addition, DNMT3A mutants harbor dominant-negative effects, such as those exhibited by DNMT3A R882H (R878H in mouse) mutated protein against wild-type DNMT3A [69, 70]. DNMT3A mutations also disrupt hematopoiesis. Researchers have used a bone marrow transplantation mouse model to determine that the function of Dnmt3a mutants in blood cell production is aberrant [37]. Additional studies have shown that mutated DNMT3A disrupts normal hematopoiesis and promotes the transformation to malignant cells, in combination with other epigenetic regulators [71]. In Dnmt3a-mutated models, a double-hit is essential for clonal expansion. In vivo experiments suggest that the mutation or deletion of Dnmt3a induces the development of leukemia by cooperating with oncogenic factors, such as *RAS* mutation, *c-Kit* variation, or FLT3-ITD abnormalities [39–43, 72]. DNMT3A mutations may also play an important role in tumor metastasis. For example, DNMT3A mutant leukemia cells may undergo leukemic extramedullary infiltration in NOD/SCID mice, a result partially linked to high expression levels of TWIST1, an epithelial-mesenchymal transition (EMT) inducer [73]. In summary, *DNMT3A* mutation exerts a great influence in hematological malignancy. A variety of small molecule compounds targeting relevant epigenetic disruptions have been developed and applied in the treatment of leukemia, which also provide a comprehensive innovation for the study of pathogenesis and targeted therapy of solid tumors.

## Conclusions

Owing to advances in sequencing technologies, numerous gene alterations associated with epigenetics have been identified in cancer genomes. Furthermore, whole-epigenome approaches, including array-based methylation profiling and bisulfite sequencing, afford a comprehensive view of the tumor methylome, and potential mechanisms of epigenetic disruption caused by DNMT changes have been explored. However, the effects of DNMT aberrations in the promotion of tumorigenesis are not entirely clear, and novel strategies for relevant targeted therapies must be developed. Future work should focus on the elucidation of tumorigenic mechanisms induced by defective DNMTs and the production of effective therapeutic approaches.

## Abbreviations

AML: Acute myeloid leukemia
BMT: Bone marrow transplantation
CGIs: CpG islands
CIMP: CpG island methylator phenotype
CTCF: CCCTC-binding factor
DNMTs: DNA methyltransferases
EMI: Extramedullary infiltration
EMT: Epithelial–mesenchymal transition
ETP-ALL: Early T-cell precursor acute lymphoblastic leukemia
HMTs: Histone methyltransferases
HSCs: Hematopoietic stem cells
LSCs: Leukemia stem cells
MDS: Myelodysplastic syndrome
MeDIP: Methylated DNA immunoprecipitation
Pre-LSCs: Pre-leukemia stem cells
SAM: S-adenosyl-l-methionine
TSGs: Tumor suppressor genes
